# Retraction: Purification and characterization of novel isoforms of the polyphenol oxidase from *Malus domestica* fruit pulp

**DOI:** 10.1371/journal.pone.0324300

**Published:** 2025-05-12

**Authors:** The PLOS One Editors

The *PLOS One* Editors retract this article [1] following concerns about peer review integrity, authorship, and concerns about irregularities in and between the published results and the underlying data provided for editorial review. We regret that the issues were not addressed prior to the article’s publication.

MT agreed with the retraction. NS, MSA, RTM, MJA, SI, AA, AR, and DA either did not respond directly or could not be reached.
